# Contour Fillings

**Published:** 1874-02

**Authors:** C. M. Wright

**Affiliations:** Ex-Professor of Mechanical Dentistry & Metallurgy in Ohio Dental College


### DENTAL REGISTER.

Vol. XXVIII.] **FEBRUARY, 1874.** [No. 2.

### CONTOUR FILLINGS.

*Read before the American Dental Society of Europe, Nov. 27th, 1873, in Basle.*

BY C. M. WRIGHT, D. D. S., EX-PROFESSOR OF MECHANICAL DENTISTRY & METALLURGY IN OHIO DENTAL COLLEGE.

Mr. President and Gentlemen :—During the cholera epidemic in Cincinnati in 1866–7, the Cin'ti Academy of Medicine, I was told, voted on the question—"Is cholera con tagious ?" The pros and cons were about equal, and a vigorous discussion ensued, which lasted perhaps six months. All the arguments on each side that experience could produce, were presented. The question was viewed in all lights, and sifted and ventilated, till every member felt so well acquainted with the "cholera question," that the dread disease itself was glad to escape from a city where it been so thoroughly exposed by so learned and scientific a body. At the end of the discussions, it was proposed that a vote should be taken on the old question ; and, strange as it may seem, the pros and cons were, as before, equally divided, and the same voices answered "aye" or "no" that had, more timidly perhaps, done the same thing six months before.

The agitated subject of a practical nature, now before our profession, is "the contour filling question," and our reports of societies from the North, South, East and West have each a place for this question. Papers are read about it. Our periodicals are spiced with it. It is discussed in operating room and laboratory, whenever two brothers meet. Like Tom Paine's "Age of Reason," it almost goes down among the maids in the kitchen and the boys in the stable. In the operative department, or rather the department of legitimate dentistry, (now so called,) this is evidently the question of the day. What the result of these discussions will be, who can tell?

Before the Mississippi Valley Society and the Mad River Society I have entered feeble protests—they were as strong ones as I could muster, however—against the indiscriminate contour operations. I tried ridicule and earnestness, and the way a certain gentleman of the West "went for that heathen—" professor, shows that I was misunderstood. Because a man can not vote aye and no on a question, and can't very well combine these into an explanation that will mean aye and no, therefore was I misunderstood.

As our Executive Committee have given us this subject for this meeting, I am compelled, by the same old "inspiration," the same old internal power, to come out again, and enter protest against the indiscriminate performance of the operaion known as the *contour*. First, because it does not, as some say, restore expression when employed in front teeth. The "walking advertisement" of a great lump of gold in labial surfaces or approximal and incising portions of incisors or cuspids, disfigures, mars, changes. affects unpleasantly the natural expression of the mouth and face, and is certainly a misfortune, and should be so regarded by dentist and patient. In this class of teeth the operation is simple, and quite apt to be undertaken by young dentists; for the coffer dam and mallet make the operation one of comparative ease.

What shall we do, then, when these teeth are considerably disorganized—when the patient has waited too long—when the teeth are badly broken down? Answer: Regard the neglect of the patient as his crime or misfortune, operate to to conserve the teeth but with the view of hiding your own work as much as possible. Let your art vision extend beyond the tooth operated on, and include the whole face, the play of the muscles, the expressions a higher artist has given your patient, and do not interfere with the original design more than the conservation of the tooth demands. "But," you say, "his own neglect has already caused gaps or spaces, that affect the original expression." My dear brother, you are not responsible for this. Because he has neglected his duty, neglected his body and induced disease, shall you act as hangman, and punish him, by increasing and changing the disfiguration?

The extreme cases of this kind, where the pulps are devitalized, and a large portion of the crown is missing, can be much better treated by employing the porcelain artificial crown and pivot. If you are not familiar with this important operation, it will well repay you to study and practice it until you are. Of course, we can not hide all our gold, all our work. I do not claim this; but I do insist that we must be conservative in this operation, if we wish to do the best for our patients and our profession.

I do not wish to be classed with those men who sacrifice teeth to insert their own china ware; for I think there are thousands who call themselves dentists, and a few who are recognized by the profession, who would, if law and justice prevailed, be to-day serving apprenticeship to shoemaking or some other harmless trade, in some of our state penitentiaries, for some of their crimes of malpractice—their wicked sacrifice of teeth and roots, their villainous treachery to God and nature, and the injury they do to the unsuspecting and ignorant people who trust them.

Thus far, I have only referred to the teeth that are plainly seen, the incisors and cuspids, and have only spoken against the contour operation as affecting expression and appearance. Shall we, or shall we not, employ the operation on bicuspids and molars? In answer to this I say: Not indiscriminately. If a tooth has decayed and become broken down on account of proximity to another tooth, and because it has been difficult for a careful or, more likely, careless patient to keep the surfaces clean,—shall we restore this objectionable shape, and leave the proximal space as before, a receptacle for deleterious agents, and out of the reach of an assured cleanness? I must say, I can not see why judgment is given us, if we don't use it better.

The perfect contour filling will in hundreds of cases leave the teeth in this condition and position. I am glad to see the conservative, the discriminative opinion gaining ground in America, and such men as Arthur, Cushing, McKellops and others standing in this new advance, which is true advance. It is a position which has observation and experience as the foundation. It is not mere opinion. I could change the words of Dr. Morgan, of Nashville, a little to suit my purpose here. A few years ago, in the American Dental Association, when the subject of soft or adhesive foil was before the house, he said: "Opinion is fact: soft foil has preserved more teeth than adhesive; for it has been known and used much longer, etc." So flat fillings have preserved more teeth than contour; for they have been known much longer, and should only be set aside and condemned after very cautious inquiry and some experience.

"Shall we never make contour operations?" Certainly. There are cases where it must be for the best; for instance, where a bicuspid or a molar stands alone and is badly broken down by disease, and this tooth can be made more useful in its work of mastication by restoring the whole of its original shape, or even extending it in length and breadth, so that this one tooth may keep in healthy exercise one or two in the opposite maxillary arch,—then, certainly, a contour filling in the best style of art is demanded. Or where a tooth is useful in sustaining a clasp, it is often better to restore the contour perfectly. Again, if there are wide spaces between the teeth and, from accident or former bad arrangement, such as decaying temporary teeth, or from general disease and neglect of the mouth, these teeth have become badly affected by caries, then probably contour operations are indicated. Then when teeth have been affected with abrasion of their grinding surfaces and are too short to perform their intended office, they may be restored with gold. In old people's mouths or in tobacco chewers' mouths, where the back teeth have been worn away and the front teeth are from an unnatural occlusion forced forward, the back teeth can be lengthened to relieve the others and restore them in health. Contour fillings can be made in these cases with an assurance of permanency.

I do not wish to discourage fine or expensive operations, when an enlightened judgment decides that they are necessary; but I do object to our profession's running wild over "mouth jeweling." It is far from a professional standard, to simply build up teeth; or make gold corners in conspicuous places, where utility and taste do not require them, or, rather, where the sternest and most reasonable necessity does not demand the operation. Shall a surgeon perform a brilliant and difficult operation, when unnecessary, or where a simple incision or good advice would be best for the patient? Shall we as dentists cut away or bur away tooth substance to get sound foundations for gold cusps or parts of teeth, when the file or chisel will remove the disease and conserve the teeth?

Dr. Atkinson wrote in 1869 the following:

"Legitimate dentistry consists in the preservation of the natural teeth in a healthy state to the end of life."

This is a good definition, and should be inscribed on the tablets of every dentist's memory; or perhaps it would be better, as some dentists have no memory, (that is of any practical use,) to have the definition printed in good form, framed and hung as a motto in every operating room.

In order to follow this legitimate dentistry, we must have the teeth kept *clean;* and, when we make operations on the teeth, I think the advance (I mean the advance who are *before* the advance of 1869) will say: "Leave all surfaces of the teeth so that they can be readily examined, and, as far as possible, leave them with self-cleaning or easily cleansed surfaces." This you see cannot be done by the universal contour maker—the man who pries apart the teeth with sticks of wood, fills the cavities with gold, restoring the integrity of the tooth, removes his wedge, and allows the same old enemy to attack the teeth in the same old way.

Away over here in Europe, we are making our reputations as Americans and a reputation for American dentistry. We can not afford to accept mere opinions or mere fancies. We have to be careful, and circumspect, and conservative, and only in the *advance* when its position is well assured. We have not come here to set diamonds in the teeth, but to conserve the teeth of those who appreciate our best efforts. We must do our work in an earnest, conscientious manner and labor rather to save teeth for the many than restore the shape of teeth for the few who might have a taste (if *taste* it may be called) for the display of gold in their mouths. Above all things, gentlemen, knowledge of conditions presented, discriminating powers cultivated by daily use, and faithfulness to our calling, great or humble though it may be considered, must preside over our manipulative abilities.

